# Editorial: The physiology, molecular biology and biochemistry in ripening and stored fruit

**DOI:** 10.3389/fpls.2023.1296816

**Published:** 2023-09-29

**Authors:** Chunhong Li, Shifeng Cao, Zhenfeng Yang, Christopher B. Watkins, Kaituo Wang

**Affiliations:** ^1^ Institute of Fruit Function and Disease Management, Department of Public Health and Management, Chongqing Three Gorges Medical College, Chongqing, China; ^2^ College of Biological and Environmental Sciences, Zhejiang Wanli University, Ningbo, China; ^3^ Horticulture Section, School of Integrative Plant Science, College of Agriculture and Life Sciences, Cornell University, Ithaca, NY, United States; ^4^ College of Biology and Food Science, Chongqing Three Gorges University, Chongqing, China

**Keywords:** fruit, ripening, chilling injury, phytohormone, transcription factor

## Introduction

The ripening process of fruits is complicated and coordinated and is characterized by obvious changes in pericarp color (green color loss and non-photosynthetic pigment increases), flesh texture (modulating by cell wall-degrading enzymes), taste (variation of sugars and organic acids), and aroma flavour (volatile productions) of fleshy fruits (Giovannoni, 2001). Specifically, most horticultural fruits of tropical origin suffer from physiological injury when exposed to cold temperature (Wang, 1989). A comprehensive understanding of the mechanism of phytohormone-mediated protection and the transcriptional regulation network may provide strategies to maintain fruit quality and reduce cold stress-induced losses. Hence, efforts to unravel the mechanisms of alleviation in fruit ripening and mitigation in chilling injury (CI) are pivotal contributing solutions to regulate postharvest fruit quality. Eleven original research articles that focus on the regulatory mechanisms of fruit ripening and chilling injury or offer effective strategies to retard fruit ripening and alleviate chilling injury were included in this Research Topic.

## Quality formation and ripening regulation of horticultural fruits

The material basis and regulator underlying fruit development and ripening still remain challenges. Teixeira et al. showed that the exocarp of green and mature grape berries is rich in chloroplasts, and they applied proteomic analysis of chloroplasts from the two phases. The authors observed that proteins associated with the Calvin cycle were stimulated in green berries, while those related to energy-generating metabolism were enriched in mature berries. Wang et al. systematically identified a batch of lignin biosynthesis-related genes and constructed a co-expression network of these genes via weighted gene co-expression network analysis. Specifically, they found the major lignin biosynthesis genes involved in ripening process and stress resistance in banana. Liu et al. reviewed NAC transcription factors, which show extensive participation in fruit yield and quality and regulate fruit ripening by directly acting on critical genes related to the biosynthesis and signal transduction of the plant hormones abscisic acid (ABA) and ethylene (ET). These articles provide the basis for the improvement of fruit development and ripening.

Postharvest ripening and senescence restrict the shelf life of horticultural crops. Both Huang et al. and Jiang et al. reported that salicylic acid (SA) treatment could maintain the organoleptic quality and postharvest storability of pummelo and blueberry. Li et al. recorded that pear had a greasy coating and yellowing process during postharvest storage, while 1-methylcyclopropene (1-MCP) could decrease the wax content of postharvest pear and delay the development of peel greasiness and yellowing by suppressing the transcription of a series of cluster genes associated with ethylene synthesis, ethylene signal transduction, wax accumulation, and chlorophyll degradation. Choi et al. investigated the molecular details of kiwifruit ripening using ethylene and its action inhibitor 1-MCP. Through a time-course transcriptomic analysis, they found that the genes related to ET synthesis and signalling suffered from opposite influence from postharvest application of 1-MCP and ET, conversely, in the process of kiwifruit ripening. They identified that the ET transcription factor *AcEIL* might exhibit an essential function in ET-induced kiwifruit ripening. These articles suggest a practical foundation for improving the protective mechanisms in relation to fruit ripening and senescence.

## Chilling injury alleviation in horticultural crops

Sensitivity to chilling can influence plant growth in the field and storability and quality during the postharvest storage period (Morris, 1982). Accordingly, the recognition of the phenomenon and the investigation of the improvement measures of chilling injury are of concern. Lin et al. compared two cultivars of hardy kiwifruit that have high frost hardiness and show similar trends in antioxidant capacities and nutritional compounds. The authors noted that the antioxidant capacity of the two hardy kiwifruit cultivars decreased but glucose increased progressively during maturation, in which the conversion from starch to total sugar was dominantly due to the expression of sucrose phosphate synthase (SPS) and fructokinase (FK). The predominant acids in the two hardy kiwifruit cultivars were quinic acid and citric acid from the early developmental to late maturity stages, respectively. These findings are conducive to a wider understanding of the physiological and biochemical basis of hardy kiwifruit for the cultivation of chilling-tolerant cultivars.

Lin et al. assessed the effect of fucoidan application on cold storage quality, reactive oxygen species (ROS) homeostasis, and energy metabolism in cucumber fruit. The authors determined that the optimum concentration of coated fucoidan was 15 g/L, which could increase DPPH and -OH scavenging rates and reduce H_2_O_2_ accumulation. The authors suggested that the improved chilling tolerance in cucumbers with fucoidan treatment may be related to the increased antioxidant enzyme activities and ROS scavenging rates, as well as high levels of ATP, ADP, and energy charge. In accordance with the effects of fucoidan in cucumbers, Zhou et al. confirmed that γ-aminobutyric acid (GABA) could lighten CI symptoms in peach fruit and that the reduction efficiency of GABA on chilling injury was associated with the accumulation of ascorbic acid (AsA) and glutathione (GSH) contents and the amplified expression profiles of AsA-GSH recycling-related genes. Moreover, the authors indicated that several ERF transcription factors, which are potentiated by GABA treatment in peach fruit, regulate AsA and GSH contents to reduce chilling injury. Overall, the authors proposed potential strategies of fucoidan coating and GABA immersion in alleviating CI symptoms in postharvest horticultural crops.

## Genetic improvement of cold tolerance in horticultural crops

In general, microRNAs (miRNAs) are a class of small, non-protein coding RNA molecules that function as negative regulators of target gene messages (Hinske et al., 2010), and their negative modulations on target genes are essential for enhancing cold tolerance (Zhao et al., 2022). Xing et al. investigated the mechanism of action of Sly-miR171d on chilling injury in tomato fruit. They found that down-regulated Sly-miR171d promotes GRAS24 expression, which obviously increased gibberellin production and C-repeat binding factor (CBF) expression and maintained cell membrane stability, therefore enhancing the chilling tolerance of tomato fruit. This study sheds light on the genetic improvement of postharvest tomato to chilling injury.

In summary, the articles in this Research Topic provide advanced information on ripening and chilling injury alleviation in horticultural crops. New insights into phytohormones, transcription factors and epigenetic modifications will impel our future applied research in the alleviation of crop ripening and chilling injury.

## Author contributions

CL: Conceptualization, Writing – original draft. ZY: Supervision, Validation, Writing – review & editing. SC: Writing – review & editing. CW: Supervision, Writing – review & editing. KW: Conceptualization, Writing – review & editing.
